# Supplementary material for: Global methane footprints growth and drivers 1990-2023
Source: Nat Commun. 2025 Sep 3;16:8184. doi: 10.1038/s41467-025-63383-5 (PMC12408836; doi:10.1038/s41467-025-63383-5)
Supplement: Supplementary file 1 — Supplementary Information [file 41467_2025_63383_MOESM1_ESM.pdf]

Supplementary Information for

Global methane footprints growth and drivers 1990-2023

Yuli Shan[1,2], Kailan Tian[3,4*], Ruoqi Li[1,5], Yuru Guan[1], Jiamin Ou[6], Dabo Guan[7,8], Klaus Hubacek[5]**

1. School of Geography, Earth and Environmental Sciences, University of Birmingham, Birmingham B15 2TT, UK
2. Birmingham Institute for Sustainability and Climate Action (BISCA), University of Birmingham, Birmingham B15 2TT, UK
3. State Key Laboratory of Mathematical Sciences, Academy of Mathematics and Systems Science, Chinese Academy of Sciences, Beijing 100190, China
4. Department of Land Economy, University of Cambridge, Cambridge CB2 1RX, UK
5. Integrated Research on Energy, Environment and Society (IREES), Energy and Sustainability Research Institute Groningen, University of Groningen, Groningen 9747 AG, the Netherlands
6. Department of Sociology, Utrecht University, Utrecht 3584 CC, the Netherlands
7. Department of Earth System Science, Ministry of Education Key Laboratory for Earth System Modelling, Institute for Global Change Studies, Tsinghua University, Beijing 100084, China
8. The Bartlett School of Sustainable Construction, University College London, London WC1E 6BT, UK

* These authors jointly supervised this work: y.shan@bham.ac.uk (YS), k.tian@amss.ac.cn (KT), k.hubacek@rug.nl (KH)

Global map for five aggregated regions

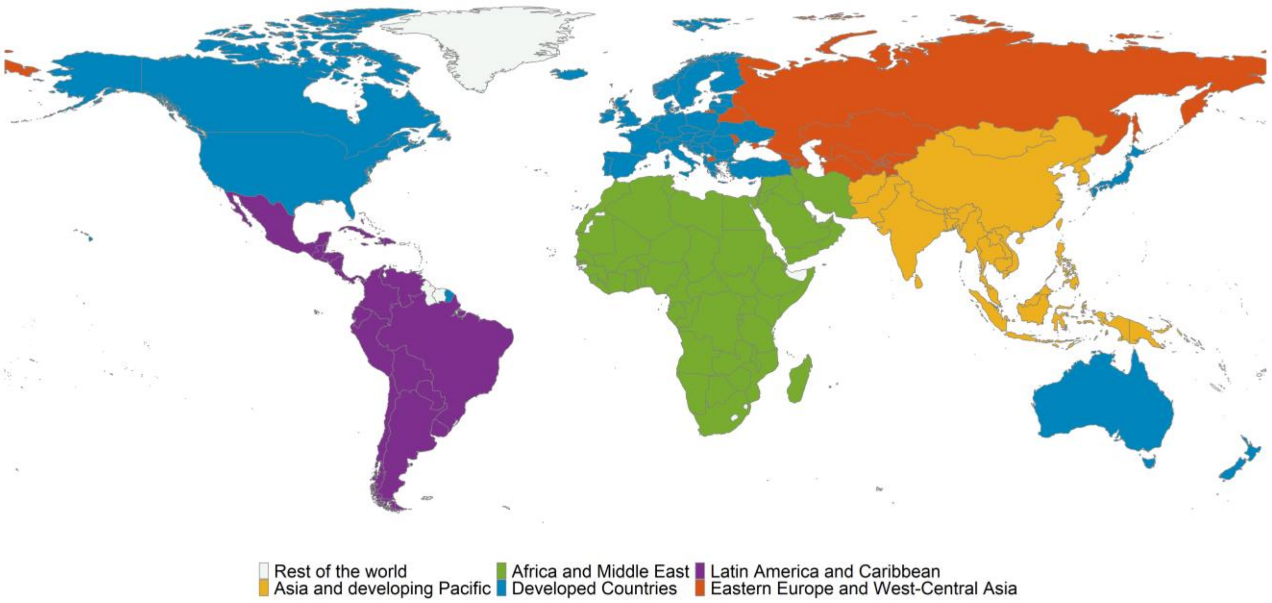

***Supplementary Fig. 1 Global map for five aggregated regions**. Country group classifications are based on the regional definitions outlined in the Intergovernmental Panel on Climate Change (IPCC) Sixth Assessment Report (AR6) [1]. The basemap layer is derived from Runfola, D. et al. geoBoundaries: A global database of political administrative boundaries. PloS one 15, e0231866 (2020), published under the CC BY 4.0 license (https://creativecommons.org/licenses/by/4.0/).*

Uncertainty and robust analysis of global methane footprints

Method

Uncertainty assessment is an important tool in emission accounting that helps assess the robustness

of emission estimates and facilitates efforts to improve their accuracy [2,3]. The uncertainties of production-based emissions (PBE) can easily be assessed with Monte Carlo simulations based on the variances of emission factors and activity data [4]. However, estimating the uncertainties of consumption-based emissions (CBE) is a more complex task as the uncertainties arise not only from the PBE coefficient but also from input-output data [5,6]. There are a number of studies investigating uncertainties of CBE. For example, Schulte, et al. [7] applied Monte Carlo simulations to analyze how uncertainty from raw data propagates to PBE. They also showed that the uncertainty can further propagate to CBE via inter-country input-output linkages. Rodrigues, et al. [8] proposed that the propagated uncertainties from PBE to CBE can be analyzed using available individual multi-regional input-output (MRIO) datasets. Therefore, in this study, we use three alternative PBE accounts (EDGAR [9], GLORIA and GTAP satellite dataset) and two alternative MRIO datasets (GLORIA [10] and GTAP [11]) to check the uncertainties of CBE, and we conclude that we derive robust results. Detailed results of each country are shown in Table S4.

Uncertainty and robustness

We first compare the methane PBE estimates from EDGAR, GLORIA, and GTAP in **Supplementary Fig. 2**. The Pearson correlation coefficients (R) displayed in each plot provide a quantitative measure of the agreement between the estimates, where values closer to 1 indicate stronger correlations. The comparison between EDGAR and GLORIA datasets shows the highest Pearson R value of 1.000, indicating a strong correlation. This suggests that the GLORIA PBE are highly consistent with EDGAR data. In contrast, when comparing GTAP data with GLORIA and EDGAR, they exhibit a little lower Pearson R value of 0.934 and 0.936, respectively. These values still represent a high correlation but suggest a little greater uncertainty and variability in the GTAP data compared to EDGAR and GLORIA. The increased scatter of points around the 1:1 line in the middle and right plots, particularly in the mid-range values, also indicates more pronounced deviations between GTAP and the other two estimates.

Overall, **Supplementary Fig. 2** demonstrates that GLORIA's PBE are of high quality, showing excellent consistency with the trusted EDGAR dataset. This highlights the reliability of using GLORIA as a robust source for PBE coefficients in accounting for countries' methane CBE in this study.

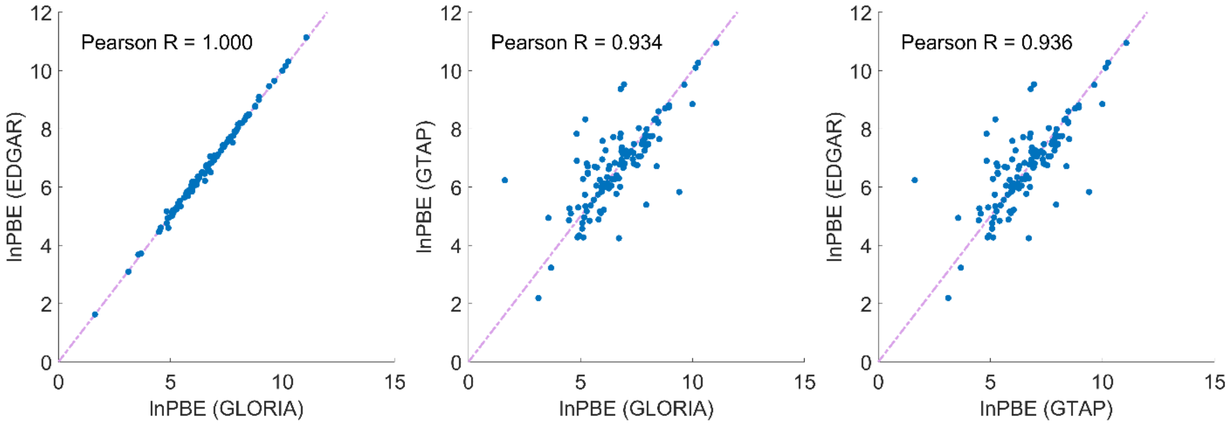

Supplementary Fig. 2 Uncertainties of production-based methane emissions (PBE) in 2014. *This figure compares per capita production-based methane emissions (PBE) across three emission datasets: GLORIA, EDGAR, and GTAP. Each panel*

presents a pairwise comparison of log-transformed PBE (lnPBE) values at the national level. The x- and y-axes represent the lnPBE values from the two datasets being compared. Each dot corresponds to a country. The dashed diagonal line indicates the 1:1 reference line, and the Pearson correlation coefficient (R) is reported in each panel to quantify the consistency between datasets.

Supplementary Fig. 3 compares our CBE (using GLORIA satellite emission data and MIRO table, on the x-axis) with alternative estimates from various sources, including EDGAR, GTAP and different configurations of GLORIA (on the y-axis). Overall, **Supplementary Fig. 3** suggests that our CBE are consistent with other estimates using alternative sources of PBE coefficient and MRIO tables. The Pearson R value ranges from 0.952 to 0.998 for different comparisons.

The three plots in the upper row of **Supplementary Fig. 3** use the GLORIA MRIO table with different sources of PBE coefficient, while the three plots in the bottom row use the GTAP MRIO table. Comparing the plots horizontally, we find that this study's estimates are highly consistent with those using GLORIA and EDGAR PBE coefficients (with R values ranging from 0.993 to 0.998). Estimates using GTAP PBE coefficients show relatively higher discrepancies with our estimates, with a noticeable reduction in the correlation (e.g., R = 0.952 and R = 0.967).

Using a different MRIO table will increase the discrepancy in CBE estimates, but to a lesser extent than using alternative PBE coefficients. For example, using the GTAP MRIO table reduces the R value to 0.993, while using alternative PBE coefficients reduces the R value to as low as 0.967, indicating that PBE coefficients carry greater uncertainty than MRIO tables.

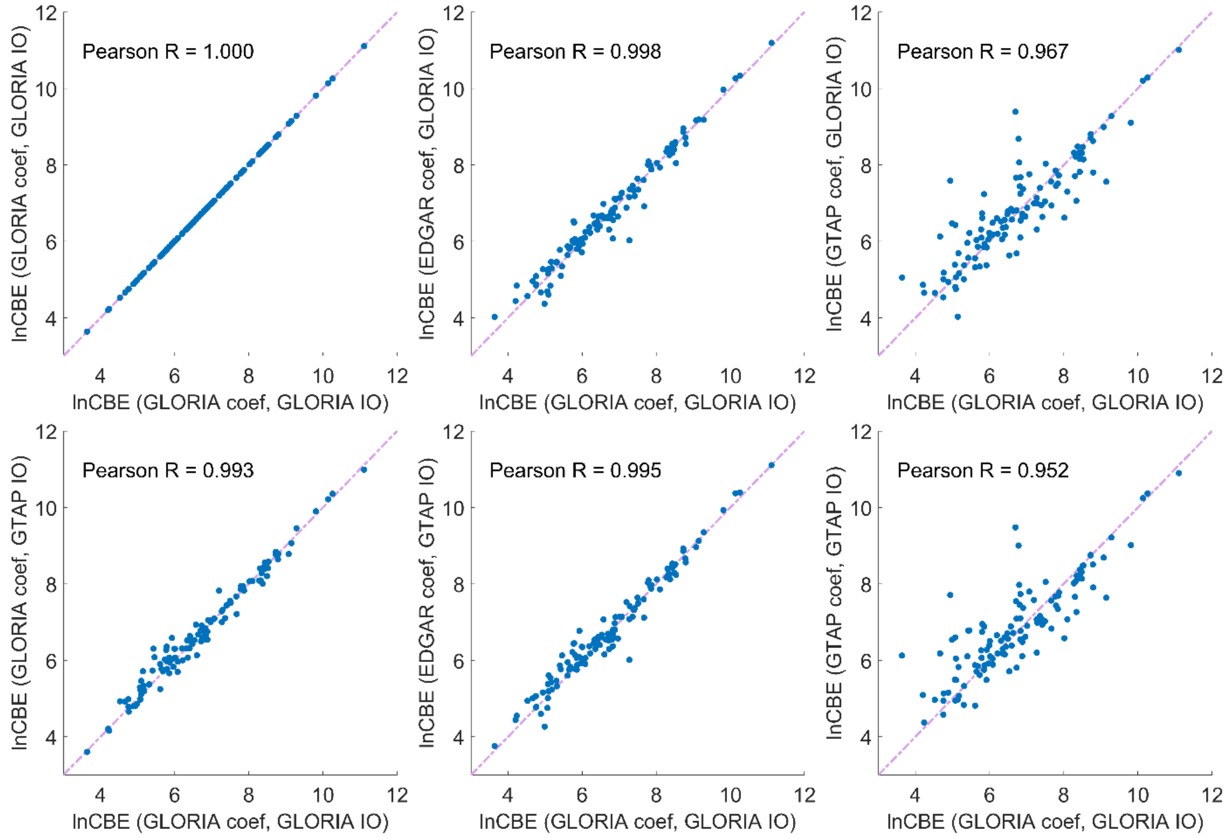

Supplementary Fig. 3 Uncertainties of consumption-based methane emissions (CBE) in 2014. *This figure compares our calculated per capita carbon-based emissions (CBE), which use GLORIA satellite-derived emission coefficients and the GLORIA input-output table (x-axis in all panels), with alternative estimates using EDGAR, GTAP, and different IO tables (y-*

axis). Each dot represents a country. The dashed diagonal line denotes the 1:1 reference line, and Pearson correlation coefficients (R) are shown in each panel to indicate consistency across datasets and methods.

Supplementary Fig. 4 further displays the time-series CBE for top countries, estimated from alternative data sources. The discrepancies between sources provide insights into the uncertainties associated with different datasets over the observation period. For most countries, such as China, India, Indonesia, Brazil, and Japan, the estimates using the EDGAR PBE coefficient (shown in red lines) tend to be slightly higher than those from GLORIA (blue lines). The inclusion of additional estimates from GTAP PBE coefficient and MRIO tables (both using GLORIA and GTAP MRIO table configurations) adds further uncertainties, shown in dots. Despite these differences, the overall trend in emissions over time is consistent between the sources, suggesting that while there are uncertainties in the absolute values, the directional changes in emissions are comparable, indicating that our estimates and analysis of countries' emission trends in the main text are robust.

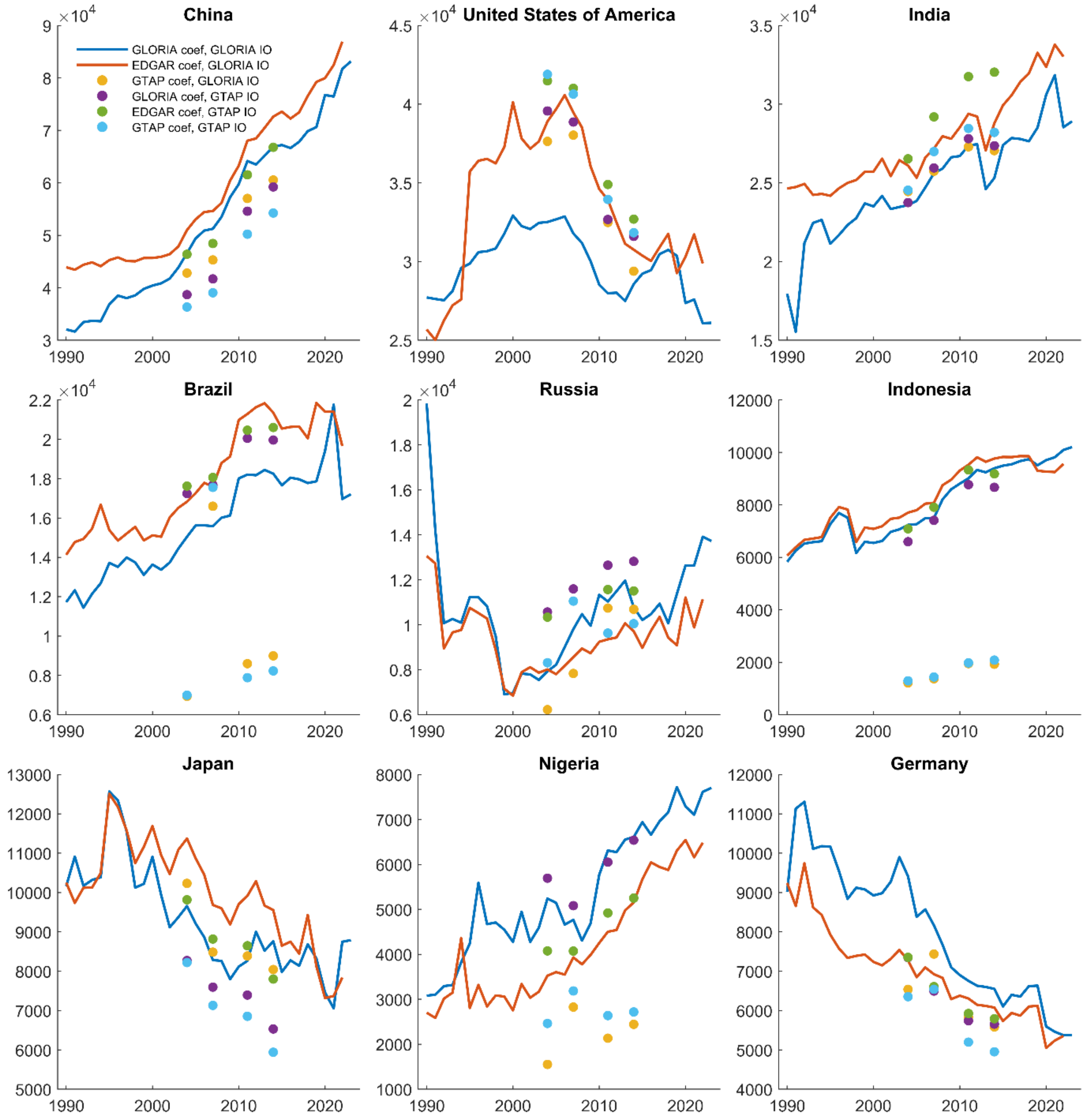

Supplementary Fig. 4 Range of methane emission footprints of top 9 countries from 1990 to 2023. *Time-series CBE*

estimates for major emitters using different emission coefficients and MRIO tables. Solid lines show results from GLORIA MRIO tables; dots represent additional estimates using GTAP MRIO tables.

Emission drivers of five top-emitting countries

Supplementary Table 1 below presents the contributions of each emission driver in selected top-emitting countries, with global results provided for comparison. The effects of these drivers exhibit greater variability at the country level compared to regional aggregates, which are shown in Figure 5 of the main text. For instance, between 2018 and 2023, the ‘emission coefficient’ driver reduced China’s emissions by 65% and India’s by 47%, even though both countries belong to the Asia and Developing Pacific region. Overall, the ‘emission coefficient’ driver decreased emissions across countries, while drivers including 'production technology’, ‘final demand per capita’, and ‘population’ generally increased emissions (with a few exceptions), that align with global trends. The driver of ‘final demand structure' exhibited more significant variations across different countries and time periods.

Supplementary Table 1 Methane emission drivers of top-emitting countries, in million tons

Country	Period	Emission coefficient	Production technology	Demand structure	Demand/capita	Population	Emission changes
Global	1998-2003	-75.48	22.07	-1.21	53.29	16.43	15.11
	2003-2008	-349.14	62.30	60.03	237.04	18.13	28.35
	2008-2013	-412.09	145.82	154.95	110.95	18.73	18.37
	2013-2018	-116.16	18.27	71.50	23.97	18.61	16.20
	2018-2023	-272.61	-56.23	234.38	100.85	13.06	19.46
China	1998-2003	-6.40	-0.76	-8.93	19.67	1.51	5.08
	2003-2008	-32.66	-0.49	-6.00	46.60	1.33	8.78
	2008-2013	-43.70	4.60	-1.83	50.80	1.67	11.54
	2013-2018	-7.26	-0.96	-11.57	24.38	1.92	6.51
	2018-2023	-45.67	-2.20	24.60	34.94	0.50	12.17
India	1998-2003	-106.07	6.47	93.03	5.37	2.06	0.87
	2003-2008	-18.85	1.52	-1.84	19.64	1.89	2.37
	2008-2013	-5.77	4.26	-9.47	8.25	1.71	-1.01
	2013-2018	-4.72	1.39	-3.04	7.83	1.54	3.00
	2018-2023	-12.96	-0.38	4.55	7.85	1.15	0.21
USA	1998-2003	-11.74	5.23	0.00	7.02	1.63	2.14
	2003-2008	-23.73	11.43	2.49	7.35	1.56	-0.90
	2008-2013	-246.35	91.58	147.44	2.11	1.18	-4.05
	2013-2018	1.45	-1.90	-2.27	5.04	1.01	3.33
	2018-2023	-19.06	-5.23	10.03	8.31	0.68	-5.27
Brazil	1998-2003	1.22	1.09	5.76	-8.18	0.92	0.82
	2003-2008	-19.33	0.76	0.42	19.19	0.82	1.85
	2008-2013	-7.67	2.16	0.52	6.62	0.80	2.43
	2013-2018	9.95	-0.13	-6.16	-5.70	0.74	-1.30
	2018-2023	-9.50	-0.60	8.88	0.43	0.50	-0.28
Russia	1998-2003	110.27	-10.51	-104.80	3.42	-0.17	-1.80
	2003-2008	-64.36	8.80	43.79	14.78	-0.12	2.88
	2008-2013	-4.03	0.87	0.53	4.32	0.06	1.75
	2013-2018	5.50	1.34	-4.45	-4.31	0.08	-1.84
	2018-2023	-62.54	-0.19	64.85	1.49	-0.01	3.60

Supplementary References

1. Dhakal, S. *et al.* Emissions Trends and Drivers. In IPCC, 2022: Climate Change 2022: Mitigation of Climate Change. Contribution of Working Group III to the Sixth Assessment Report of the Intergovernmental Panel on Climate Change. (Cambridge University Press, Cambridge, UK and New York, NY, USA, 2022).
2. Jonas, M., Marland, G., Krey, V., Wagner, F. & Nahorski, Z. Uncertainty in an emissions-constrained world. *Uncertainties in Greenhouse Gas Inventories: Expanding Our Perspective*, 9-26 (2015).
3. Intergovernmental Panel on Climate Change (IPCC). *Revised 1996 IPCC Guidelines for national greenhouse gas inventories*. (Intergovernmental Panel on Climate Change, 1996).
4. Shan, Y. *et al.* Data Descriptor: China CO_2 emission accounts 1997-2015. *Sci. Data* **5**, doi:10.1038/sdata.2017.201 (2018).
5. Lenzen, M., Wood, R. & Wiedmann, T. Uncertainty analysis for multi-region input-output models - a case study of the UK's carbon footprint. *Econ. Systems Res.* **22**, 43-63, doi:10.1080/09535311003661226 (2010).
6. Dhakal, S. *et al.* in *Climate Change 2021: Mitigation of Climate Change. Contribution of Working Group III to the Sixth Assessment Report of the Intergovernmental Panel on Climate Change* (2021).
7. Schulte, S., Jakobs, A. & Pauliuk, S. Estimating the uncertainty of the greenhouse gas emission accounts in global multi-regional input–output analysis. *Earth Syst. Sci. Data* **16**, 2669-2700 (2024).
8. Rodrigues, J. F., Moran, D., Wood, R. & Behrens, P. Uncertainty of consumption-based carbon accounts. *Environ. Sci. Technol.* **52**, 7577-7586 (2018).
9. Author. EDGAR (Emissions Database for Global Atmospheric Research) Community GHG Database-EDGAR CO_2, EDGAR CH_4, EDGAR N_2O, EDGAR F-GASES. <https://edgar.jrc.ec.europa.eu/> (Institution, 2024).
10. Lenzen, M. *et al.* Implementing the material footprint to measure progress towards Sustainable Development Goals 8 and 12. *Nat. Sustain.* **5**, 157-166 (2022).
11. Aguiar, A., Chepeliev, M., Corong, E. & van der Mensbrugghe, D. The global trade analysis project (GTAP) data base: Version 11. *Journal of Global Economic Analysis* **7** (2022).
