# Supplementary material for: Global methane footprints growth and drivers 1990-2023
Source: Nat Commun. 2025 Sep 3;16:8184. doi: 10.1038/s41467-025-63383-5 (PMC12408836; doi:10.1038/s41467-025-63383-5)
Supplement: Supplementary file 2 — Description of Additional Supplementary Files [file 41467_2025_63383_MOESM2_ESM.pdf]

Description of Additional Supplementary Files

File Name: Supplementary Data 1
Description: Production and sectoral consumption-based methane emissions of countries and groups

File Name: Supplementary Data 2
Description: Decoupling index of countries and groups

File Name: Supplementary Data 3
Description: Sectoral emissions embodied in trade of countries and groups

File Name: Supplementary Data 4
Description: Uncertainty range of consumption-based methane emissions of countries
